# Breed, parity, and days in milk affect risk of tongue rolling in dairy cows

**DOI:** 10.3168/jdsc.2022-0321

**Published:** 2023-02-09

**Authors:** J.A. Robbins, K. McCandless, D.M. Weary, M. Paros

**Affiliations:** 1 Department of Animal and Dairy Sciences, University of Wisconsin, Madison 53706; 2 Department of Biological Sciences, The Evergreen State College, Olympia, WA 98505; 3 Animal Welfare Program, Faculty of Land and Food Systems, University of British Columbia, Vancouver, BC, Canada V6T 1Z4

## Abstract

Stereotypic behaviors are repetitive, invariant movements with no obvious biological function. Tongue rolling (TR) is a common stereotypic behavior in cattle, characterized by a repeated circular movement of the tongue inside or outside of the mouth. We assessed TR in adult lactating dairy cows (from 45 to 305 d in milk; DIM) on a large commercial US dairy comprised of Jersey and Jersey-Holstein crosses (n = 8,158 cows). Cows were monitored during each of 3 consecutive milkings using video cameras located at the center of 2 rotary parlors. In total, 29.0% (2,365/8,158) of cows tongue rolled at least once, 7.9% (646/8,158) at least twice, and 1.7% (141/8,158) tongue rolled during all 3 milkings. The effects of breed (Jersey vs. Jersey-Holstein cross), parity (first lactation versus older), DIM, and the interactions between breed and parity and DIM on TR (comparing cows that were never observed rolling versus cows observed doing so at least once) were tested using logistic regression, revealing interactions between breed and parity. Among primiparous cows, Jerseys were more likely than Jersey-Holstein crosses to tongue roll [odds ratio (OR) = 1.61, confidence interval (CI) = 1.35–1.92]; similarly, among second-parity and older cows, Jerseys were again more likely to tongue roll than were Jersey-Holstein crosses (OR = 2.35, CI = 1.95–2.83). The effect of DIM differed by breed and parity; for primiparous Jerseys, the odds of TR increased with DIM (OR = 1.31, CI 1.12–1.52, for every 100-d increase), and for Jersey-Holsteins cows the odds of TR decreased with DIM (OR = 0.61, CI 0.43–0.88, for every 100-d increase). These breed, parity, and stage of lactation differences within a single farm suggest a role of both genetic and developmental effects in the proclivity to tongue roll.

## Highlights

• 29% (2,365/8,158) of cows were observed tongue rolling at least once.
• Jersey cows were more likely to tongue roll than were Jersey-Holstein crosses.
• Second-parity and older cows were more likely to tongue roll than were first-parity cows.
• Tongue rolling tended to increase in early lactation and then decline in later lactation.

## Graphical Abstract

**Figure:**
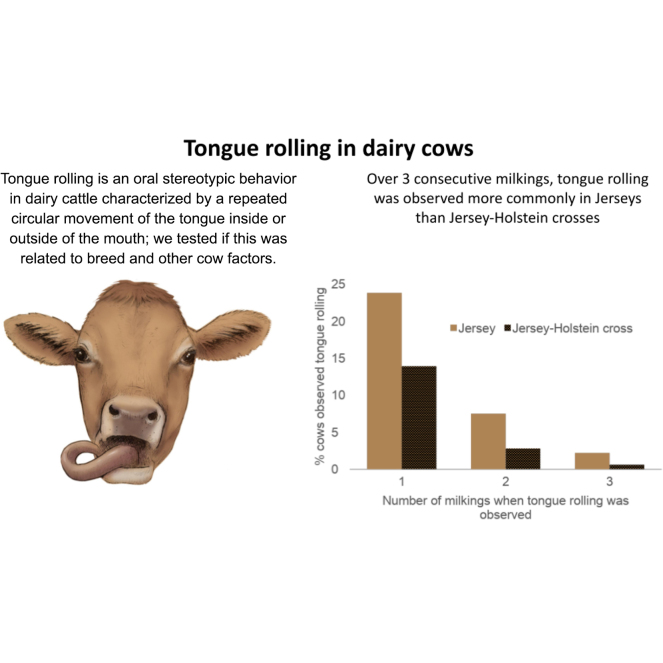
**Summary:** Tongue rolling is a common oral stereotypic behavior in dairy cattle characterized by a repeated circular movement of the tongue inside or outside of the mouth. We monitored tongue rolling in 8,158 lactating Jersey and Jersey-Holstein crosses on a large commercial dairy farm. The behavior was recorded over 3 consecutive milkings by cameras mounted in the center of two 80-stall rotary parlors. About one-third of the cows were observed tongue rolling during at least one milking, but this behavior was more common in Jerseys than in Jersey-Holstein crosses and more common in older than younger cows. We encourage more work on the stereotypic behaviors of cows on commercial farms to better understand the role of genetics and other developmental factors.

Stereotypies are repetitive, invariant behaviors with no obvious goal or function (Mason, 1991). When comparing housing conditions, the presence of stereotypic behaviors may indicate suboptimal environments and diminished welfare (Mason and Latham, 2004). Once stereotypic behaviors develop they are difficult to alter. Nonnutritive oral behaviors are the most common form of stereotypy in captive ungulates, including crib-biting in horses, sham-chewing in sows, and tongue rolling in cattle (Bergeron et al., 2006). Among cattle, nonnutritive oral behaviors may be directed at the environment, other animals (e.g., cross-sucking), or at nothing in particular as is the case with tongue rolling. Tongue rolling occurs when cattle repeatedly move their tongue in a circular pattern either inside or outside of their mouths (Redbo, 1990). These behaviors are classified as nonnutritive because they occur in the absence of feed or cud. Stereotypies may result from an inability to engage in highly motivated behaviors, and previous research on tongue rolling has focused on the effects of feeding practices (Redbo et al., 1996; Redbo and Nordblad, 1997). Restricted (vs. ad libitum) TMR feeding and reduced forage:concentrate ratios increase tongue rolling in adult dairy cattle (Redbo and Nordblad, 1997). Research using fistulated cows (to control for rumen load) found that increased tongue rolling was associated with decreased feed duration (Lindström and Redbo, 2000). This latter result suggests that tongue rolling is associated with the motivation to engage in specific feeding behaviors, although factors in addition to feeding management are also likely involved.

Webb et al. (2017) suggested a gene × environment interaction in the development of oral stereotypies and previous authors have suggested that tongue rolling is more common in Jersey cattle (e.g., Grandin, 2017), but we are not aware of any empirical data to support this claim. The objectives of this study were to describe the prevalence of tongue rolling on a large commercial farm, test the hypothesis that tongue rolling is more common among Jersey cattle (compared with Jersey-Holstein crosses), and assess the effects of parity class and DIM as cow-level factors.

All procedures in this study were approved by the Evergreen State College Institutional Animal Care and Use Committee (IACUC protocol 22–012). Data were collected in July 2020 on a large farm with Jersey and Jersey-Holstein crossbred cows, located in the Western United States. Lactating cows were housed in 19 pens grouped by DIM, milk production, pregnancy status, and parity. The mean (±SD) number of cows per pen was 526 ± 49. As calves, all animals were reared on-site under identical conditions of individual housing and milk feeding by bottle. As adults, cattle were housed in free-stalls bedded twice per week with composted dairy solids. Lactating cows were milked twice daily, at 12-h intervals, using a double-rotary, 80-stall parlor (PR 3100, DeLaval). All cows received the same TMR, delivered twice daily with feed pushups occurring every 2 h. Milking parlor observations occurred via instantaneous scan sampling for 3 consecutive milkings (during a single 36-h period) using images from cameras (TLC 200, Brinno) in the center of two 80-stall rotary parlors (PR 2100, DeLaval) directly facing cows as they entered the rotary. The frequency of observation was dictated by time constraints. Each camera was positioned to record 11 milking cows in a single frame and captured one photo every 2 s; this resulted in each cow being recorded for 64 s (32 images) constituting 14% of total milking time on the rotary. Each photo was coded for tongue rolling by a single observer (KM), recording presence or absence of tongue rolling (i.e., cows engaged in active curling and uncurling of the tongue in a rapid motion inside or outside of the mouth; Redbo et al., 1992; Webb et al., 2017). Individual cows were identified using radio frequency identification ear tags and then later synchronized with the recorded rotary stall number and milking time. Individual cow and pen identification, milking times, and milk quantity were obtained for all cows from the milk recording system (DelPro, DeLaval). Other cow-level data (breed, DIM, pregnancy status, lactation number) were extracted from farm records. Only lactating cows from 45 to 305 DIM were included in the data set. Any animals not recorded during all 3 consecutive milkings, or those with incomplete records, or subject to pen moves, were excluded, resulting in a final sample of 8,158 cows.

Statistical analyses were performed in SAS Studio (SAS OnDemand for Academics, SAS Institute Inc.) considering the cow as the unit of analysis. Parity was also dichotomized as primiparous or multiparous. Days in milk was considered as continuous, with both linear and quadratic effects considered. The full logistic regression model also included interactions between breed and parity and breed and DIM. The dependent variable (tongue rolling) was dichotomized as whether or not the cow was observed tongue rolling during at least 1 of the 3 milking parlor scans (i.e., there was only one observation/cow included in the analysis). Assumptions were verified by scrutinizing the residuals.

Breed composition was 72.8% (5,943/8,158) Jersey and 27.2% (2,215/8,158) Jersey-Holstein. Approximately 45% (3,678/8,158) of these animals were in their first lactation, and DIM (±SD) averaged 170 ± 78. In total, 29.0% (2,365/8,158) of cows were observed tongue rolling at least once over the course of the 3 observations in the milking parlor, and Jerseys were observed tongue rolling nearly twice as frequently (33.4%; 1,984/5,953) as the Jersey-Holstein crosses (17.2%; 381/2,215).

The complete logistic regression model revealed an interaction between breed and parity (*P* = 0.0022), so the effect of breed was tested separately by parity class. Among first-parity cows, Jerseys were more likely to tongue roll than were Jersey-Holstein crosses [odds ratio (**OR**) = 1.61, CI = 1.35–1.92; *P* < 0.0001]; out of a total of 2,181 primiparous Jerseys, 478 (21.9%) were observed tongue rolling at least once, in comparison to 222 of 1,497 (14.8%) Jersey-Holstein crosses. Similarly, among second-parity and older cows, Jerseys were again more likely to engage in this behavior than were Jersey-Holstein crosses (OR = 2.35, CI = 1.95–2.83; *P* < 0.0001); 40.0% (1,506/3,762) vs. 22.1% (159/718), respectively.

A visual assessment of plots showing the effect of DIM revealed differences by breed and parity class, so linear and quadratic effects were analyzed separated by breed and parity (Figure 1). For primiparous Jerseys, the odds of tongue rolling increased with DIM (*P* < 0.0178), with a tendency for a negative quadratic term (*P* < 0.0830). We also found curvilinear effects of DIM for the primiparous Jersey-Holsteins (*P* = 0.0384 for the linear term, and *P* = 0.0283 for the quadratic term), and for the Jersey cows (*P* = 0.0268 for the linear term, and *P* = 0.0213 for the quadratic term), but found no effect of DIM for Jersey-Holstein cows (*P* = 0.8822 for the linear term, and *P* = 0.7208 for the quadratic term).

**Figure 1:**
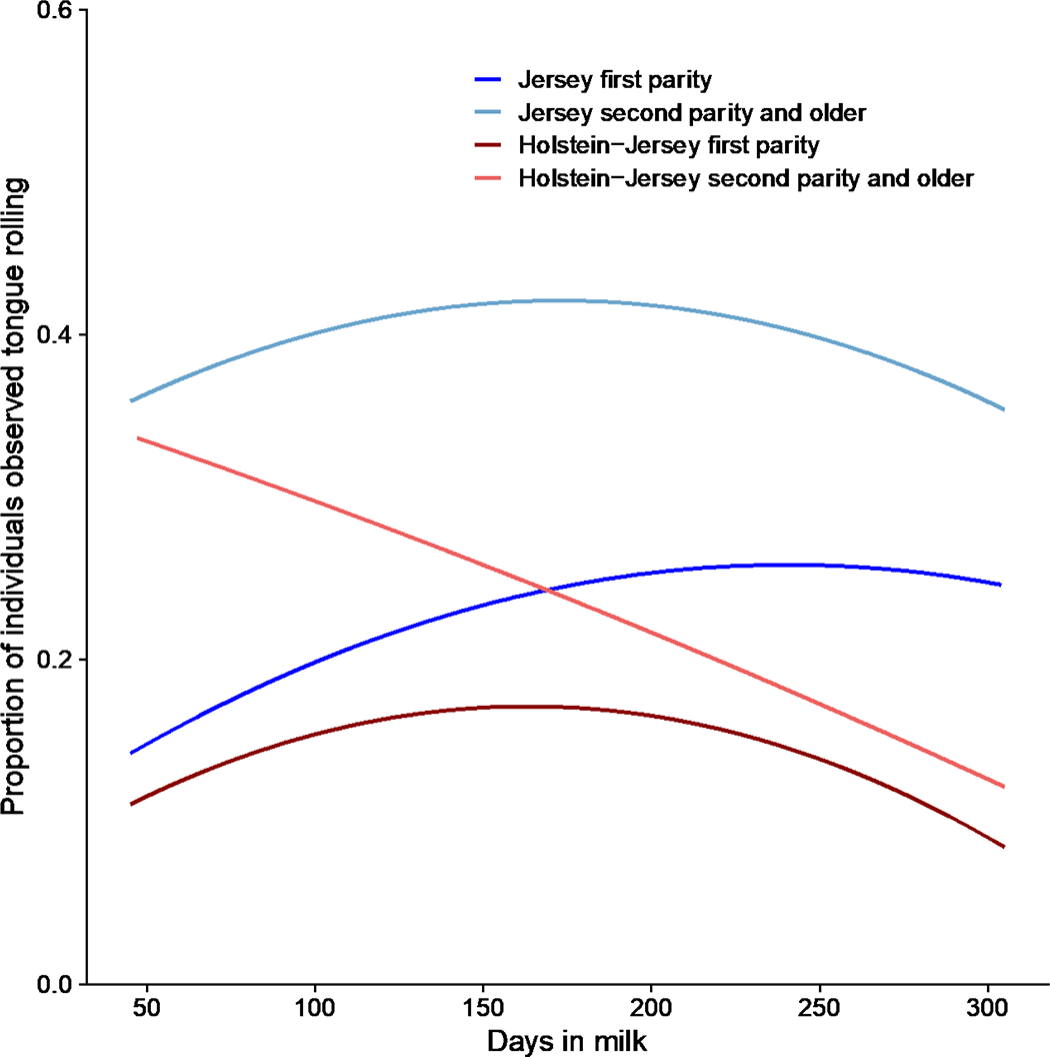
The proportion of cows (n = 8,158) observed tongue rolling while in the milking parlor, in relation to DIM, shown separately for Jersey first-parity cows (n = 2,181), Jersey second-parity and older cows (n = 3,762), Holstein-Jersey crossbred first-parity cows (n = 1,497), and Holstein-Jersey second-parity and older cows (n = 718).

A limitation of the current study is that each cow was observed only 3 times, and on each occasion for approximately 1 min while in the milking parlor. Despite this limitation, we found nearly one-third of the cows tongue rolled. More intensive work, following the same cows for more days and longer periods (and elsewhere in the barn), is required to determine how sampling method affects estimates of prevalence for this behavior. Another limitation is that the results are from a single herd. We encourage future work to assess tongue rolling in more herds, ideally in other herds with a combination of breeds. Given the important breed effects identified here, future work may wish to examine genetic effects within breed, for example attempting to identifying DNA markers associated with this behavior. Last, video coding was carried out by a single, trained observer which may have introduced observer error. Subsequent research would benefit from multiple observers and assessing inter- and intraobserver reliability (Kaufman and Rosenthal, 2009).

Two previous observational studies on 95 and 37 lactating Swedish Red and White cattle reported tongue rolling in 42% and 73% of cattle, respectively (Redbo, 1992; Redbo et al., 1996), values higher than the 29% recorded in the current study. A more recent study (Binev, 2022) found that just 4% of 1,100 Holstein lactating cattle were recorded as tongue rolling. Another study reported tongue rolling on 157 commercial veal farms in Europe and found that just 3% of calves were engaged in the behavior (Leruste et al., 2014). Differences between studies may be due to disparities in how tongue rolling was defined, environmental factors, sampling methodology, and characteristics of the animals observed. A major strength of the current study is that we were able to assess the effects of breed and DIM using a large sample of cows all managed in a consistent manner.

We found that tongue rolling was far more common in Jersey cows versus the Jersey-Holstein crosses. Previous research has either not reported breed (Ishiwata et al., 2008) or focused on Holstein (Leruste et al., 2014; Webb et al., 2017; Binev, 2022), Japanese Black (Sato et al., 1994; Seo et al., 1998), Simmental (İssi̇ et al., 2009; Fuerst-Waltl et al., 2010; Schneider et al., 2019), or Swedish Red and White cattle (Lindström and Redbo, 2000; Redbo, 1990, 1992, 1998; Redbo et al., 1996; Redbo and Nordblad, 1997). A study on European veal farms (Le Ruste et al., 2014) found a higher risk of oral manipulation of substrates in crossbred or meat-type calves compared with calves of dual-purpose breeds, but breed was not a factor in their multivariable model. Fuerst-Waltl et al. (2010) found genetic variation in the expression of cross-sucking in calves when comparing different Austrian Fleckvieh sires, but a study of Holstein, Brown Swiss, and Simmental cattle failed to find an association between breed and this behavior (Keil et al., 2001). Nielsen et al. (2008) found that Jersey calves were more likely to cross-suck penmates compared with Danish Red and Holstein calves. A higher level of tongue rolling in Jerseys may be the result of a greater motivation to graze, which involves using the tongue while eating. Future research should directly assess breed differences in motivation and how this motivation might relate to the development of tongue rolling. We urge caution when reporting prevalence of tongue rolling (and other stereotypic behaviors) without considering breed composition.

Previous research on tongue rolling has focused on feeding motivation (e.g., Ridge et al., 2020). For example, limit-fed cows were more likely to tongue roll than were cows fed ad libitum (Redbo et al., 1996), and the addition of long straw to the ration reduced tongue rolling even though energy remained constant (Redbo and Nordblad, 1997). These results indicate that feed ingestion is associated with this behavior. In the current study, all cows were fed the same TMR ad libitum. Research comparing Holsteins and Jerseys has found the latter spend more time eating TMR and ruminating (per kg of feed consumed) and feed at more regular intervals throughout the day (Aikman et al., 2008). Jerseys also spend more time masticating and chew faster when grazing than Holsteins (Prendiville et al., 2010). A higher level of tongue rolling in Jerseys may be the result of a greater motivation to graze and use their tongue while eating. Future research should directly assess breed differences in motivation and how this motivation might relate to the development of tongue rolling.

Tongue rolling was more common in older animals, especially for Jersey cows, and tended to increase from early to mid lactation for all cows except older Jersey-Holstein crosses. Although tongue rolling is likely more common as cows age, other factors (such as higher culling or death rates for nonstereotypic cows) could also have resulted in the age-related patterns we observed; longitudinal work is required to better understand this relationship. The effects of DIM may be due in part to changes in feed intake associated with milk production and gestation; further work in a facility that monitors individual feed intake is needed to better assess this relationship.

Previous research in zoo animals has reported that public awareness of stereotypic behavior leads to more negative public perceptions about animal care and decreased support for keeping animals in these environments (Miller, 2012). Given the higher rates of this behavior in Jersey cows, and the large number of Jersey herds (approximately 30% of US dairy operations; USDA, 2016), this behavior creates a potential public perception risk. Further research is required into the welfare consequences of the behavior and how any negative effects could be mitigated.
